# Impairment and Restoration of Homeostatic Plasticity in Cultured Cortical Neurons From a Mouse Model of Huntington Disease

**DOI:** 10.3389/fncel.2019.00209

**Published:** 2019-05-16

**Authors:** Amy I. Smith-Dijak, Wissam B. Nassrallah, Lily Y. J. Zhang, Michal Geva, Michael R. Hayden, Lynn A. Raymond

**Affiliations:** ^1^Graduate Program in Neuroscience, The University of British Columbia, Vancouver, BC, Canada; ^2^Department of Psychiatry, Djavad Mowafaghian Centre for Brain Health, The University of British Columbia, Vancouver, BC, Canada; ^3^Research and Development, Teva Pharmaceutical Industries Ltd., Netanya, Israel; ^4^Centre for Molecular Medicine and Therapeutics, The University of British Columbia, Vancouver, BC, Canada

**Keywords:** Huntington disease, homeostatic, synaptic, plasticity, calcium, BDNF, pridopidine

## Abstract

Huntington disease (HD) is an inherited neurodegenerative disorder caused by a mutation in the *huntingtin* gene. The onset of symptoms is preceded by synaptic dysfunction. Homeostatic synaptic plasticity (HSP) refers to processes that maintain the stability of networks of neurons, thought to be required to enable new learning and cognitive flexibility. One type of HSP is synaptic scaling, in which the strength of all of the synapses onto a cell increases or decreases following changes in the cell’s level of activity. Several pathways implicated in synaptic scaling are dysregulated in HD, including brain-derived neurotrophic factor (BDNF) and calcium signaling. Here, we investigated whether HSP is disrupted in cortical neurons from an HD mouse model. We treated cultured cortical neurons from wild-type (WT) FVB/N or YAC128 HD mice with tetrodotoxin (TTX) for 48 h to silence action potentials and then recorded miniature excitatory postsynaptic currents. In WT cultures, these increased in both amplitude and frequency after TTX treatment, and further experiments showed that this was a result of insertion of AMPA receptors and formation of new synapses, respectively. Manipulation of BDNF concentration in the culture medium revealed that BDNF signaling contributed to these changes. In contrast to WT cortical neurons, YAC128 cultures showed no response to action potential silencing. Strikingly, we were able to restore the TTX-induced changes in YAC128 cultures by treating them with pridopidine, a drug which enhances BDNF signaling through stimulation of the sigma-1 receptor (S1R), and with the S1R agonist 3-PPP. These data provide evidence for disruption of HSP in cortical neurons from an HD mouse model that is restored by stimulation of S1R. Our results suggest a potential new direction for developing therapy to mitigate cognitive deficits in HD.

## Introduction

Huntington disease (HD) is an autosomal dominant neurodegenerative disorder caused by an expansion of the CAG repeat region in the first exon of the gene encoding the protein huntingtin (Htt) (MacDonald et al., 1993). Neurodegeneration in HD begins with the death of striatal spiny projection neurons (SPNs), but is preceded by altered function at the corticostriatal synapse (Cepeda et al., 2007; Milnerwood and Raymond, 2010; Plotkin and Surmeier, 2015), and altered cortical activity and plasticity, which has been documented in humans a decade before disease onset (Cepeda et al., 2007; Schippling et al., 2009; Milnerwood and Raymond, 2010; Orth et al., 2010). It has also been found that knocking out mutant Htt (mHtt) in the cortex of HD model mice ameliorates their behavioral changes (Estrada-Sánchez et al., 2015). This suggests that, while striatal neurons are the first to die in HD, cortical neurons are also important in early HD pathophysiology.

Wild-type (WT) Htt interacts with hundreds of proteins involved in many aspects of cellular function, and the presence of mHtt disrupts a correspondingly large number of processes (Shirasaki et al., 2012; Saudou and Humbert, 2016). One of these processes which has received little attention, but which has recently been highlighted as being of potential interest (Wang et al., 2017), is homeostatic synaptic plasticity (HSP). HSP refers to a group of processes, such as synaptic scaling and metaplasticity, which maintain the level of activity of a network of neurons around a set point. Without these processes, a network which could only undergo the classical plasticity processes of long-term potentiation (LTP) and long-term depression (LTD) would eventually become either hyper- or hypoconnected; with either state resulting in neuronal dysfunction and death (Marder and Goaillard, 2006; Turrigiano, 2007; Watt and Desai, 2010). The set of genes involved in HSP overlaps with those involved in multiple neurodegenerative disorders, including several types of episodic ataxia, spinocerebellar ataxia type 19, and fragile X syndrome, as well as HD (Wang et al., 2017).

One point of overlap between HSP and HD is brain-derived neurotrophic factor (BDNF). While Htt does not directly interact with BDNF, it does interact with many proteins involved in BDNF transport and signaling, and BDNF signaling through the TrkB receptor is reduced in HD (Zuccato and Cattaneo, 2007; Milnerwood and Raymond, 2010; Shirasaki et al., 2012; Plotkin and Surmeier, 2015; Saudou and Humbert, 2016). BDNF signaling is also involved in synaptic scaling, a type of HSP. Synaptic scaling is a phenomenon wherein the strength of all of the synapses onto a neuron increases or decreases in response to a change in a neuron’s overall level of activity. In excitatory neurons, such as cortical pyramidal neurons (CPNs), increased activity results in decreased synaptic strength, referred to as synaptic down-scaling. Decreased activity results in increased synaptic strength, referred to as synaptic up-scaling (Turrigiano et al., 1998). BDNF is released in an activity-dependent manner, and it is thought that reduced activation of the TrkB receptor by BDNF is a signal for synaptic up-scaling (Rutherford et al., 1998; Turrigiano, 2007; Watt and Desai, 2010). If this signaling is disrupted, as is the case in HD, it could interfere with synaptic up-scaling.

Another notable point of overlap between HD and HSP is calcium homeostasis (Shirasaki et al., 2012; Ryskamp et al., 2017; Wang et al., 2017). Cytoplasmic calcium concentration has been shown to be increased in SPNs from HD model mice as a result of increased entry through *N*-methyl-D-aspartate receptors (NMDARs), whose subunit composition and localisation in these cells is altered (Milnerwood et al., 2010, 2012), and sensitisation of the inositol 1,4,5-trisphosphate receptor (IP3R), which results in increased release of Ca^2+^ from the endoplasmic reticulum (ER) (Tang et al., 2003; Ryskamp et al., 2017). Dysregulated cytoplasmic Ca^2+^ is thought to contribute to the early dysfunction and eventual death of SPNs in HD (Milnerwood and Raymond, 2010). Calcium signaling has been implicated in multiple types of HSP (Marder and Goaillard, 2006; Turrigiano, 2007; Watt and Desai, 2010), and elevated cytoplasmic Ca^2+^ could interfere with any of them.

Together, this suggested to us that the disruption of BDNF and calcium homeostasis in HD may interfere with HSP. The drug pridopidine, which has been investigated as a treatment for HD (Kieburtz et al., 2018), has been shown to normalize both of these signaling pathways (Geva et al., 2016; Ryskamp et al., 2017). We therefore set out to investigate whether HSP, and specifically synaptic scaling, is disrupted in cortical neurons from the YAC128 HD mouse model, and whether pridopidine could restore any such deficit.

## Materials and Methods

### Cell Culture

FVB/N WT or YAC128 (line 55) mice (Slow et al., 2003) were bred and maintained in the University of British Columbia Animal Resource Unit according to guidelines of the Canadian Council on Animal Care, under the approved protocol A15-0069. Cortical neurons were isolated from mouse pups as previously described (Milnerwood et al., 2012). If the culture was being prepared for electrophysiology, 2.7 million cells were suspended in 12 mL D minimum essential medium (DMEM, GIBCO) with 10% FBS (DMEM+). For cultures prepared for immunohistochemistry, 2 million cells were suspended in 100 μL electroporation buffer (Mirus Bio) with a plasmid encoding GFP (Addgene plasmid 37825). This solution was placed in a cuvette, electroporated (AMAXA nucleofector I: program 03), and resuspended in 12 mL DMEM+ with 0.7 million non-transfected cells. Cells were plated at a density of 1.125 × 10^5^ cells per cm^2^ in a 24-well plate. After 3 h, DMEM+ was replaced with 0.5 mL plating medium (PM; 2% B27, Invitrogen; penicillin/streptomycin; 2 mM α-glutamine; neurobasal medium, GIBCO). An additional 0.5 mL/well PM was added at 3 days *in vitro* (DIV), half of the culture’s PM was replaced at DIV10 and again 4 days before recording or fixation.

### Drug Treatment

For experiments involving pridopidine or R(+)-3-(3-Hydroxyphenyl)-*N*-propylpiperidine (3-PPP; Sigma), cells were treated with 0.1, 1, or 10 μM pridopidine (Teva Pharmaceuticals Ltd.), or 1 μM 3-PPP (Sigma) during the half-medium change 4 days before recording or fixation. To test induction of HSP, cells were treated with 2 μM tetrodotoxin (TTX; Affix Scientific) vs. dH_2_O (vehicle) with or without 25 ng/mL BDNF (Thermo Fisher Scientific), or else with 1.5 μg/mL TrkB-Fc (R&D Systems) 2 days before recording or fixation. TTX was stored and handled in accordance with the University of British Columbia’s chemical and biological safety standards.

### Electrophysiology

Miniature excitatory postsynaptic currents (mEPSCs) were recorded using an Axopatch 200B, Axon Digidata 1550B or MultiClamp 700A amplifier and pClamp 10.2, 10.3, or 10.6 software (Molecular Devices, Palo Alto, CA, United States). CPNs were identified by morphology and recordings were made at DIV 20–22. Cells were voltage clamped at -70 mV, and their intrinsic membrane properties were determined using a 10 mV hyperpolarizing pulse immediately after whole-cell access was achieved. Cells were accepted for recording if they had a holding current more positive than -500 pA and a series resistance of up to 25 MΩ, with most cells’ series resistance under 20 MΩ. mEPSCs were recorded for up to 5 min. If the series resistance changed by more than 20% during the course of the recording, or if the holding current fell below -500 pA, events were not analyzed beyond the point where this threshold was exceeded. Cells were excluded from analysis if their series resistance changed by more than 20%, or their holding current fell below -500 pA, during the first 30 s of the recording, or if they had fewer than 50 events with an amplitude of greater than 10 pA. Cells were recorded in artificial cerebrospinal fluid (ACSF) containing (mM): 167 NaCl, 2.4 KCl, 10 Glucose, 10 HEPES, 2 CaCl_2_, 1 MgCl_2_, 0.05 picrotoxin (PTX; Tocris), 0.0005 TTX (Affix Scientific), pH 7.3 with NaOH, 310–320 mOsm (all chemicals from Sigma except for PTX and TTX). The recording electrode (3–6 MΩ) was filled with an internal solution containing (mM): 145 K-Gluconate, 1 MgCl_2_, 10 HEPES, 1 EGTA, 2 MgATP, 0.5 Na_2_GTP, pH 7.3 with KOH, 280–290 mOsm. mEPSC recordings were analyzed with Clampfit 10.2 and 10.7, using its template search function.

### Peak-Scaled Non-stationary Noise Analysis of AMPAR-Miniature EPSCs

To measure mean AMPAR channel conductance (γ) and the number of channels exposed to glutamate (*N*) per synapse, peak scaled noise analysis of AMPAR-miniature EPSCs (mEPSCs) was performed using Clampfit 10.2 (Molecular Devices). The peak of each mEPSC in a recording was scaled to the average mEPSC waveform for that recording, and the variance of current around the mean for each time point was calculated. The data were fit with the following parabolic equation:

$$\sigma^{2}=il=I^{2}/N+\sigma_{b}^{2}$$

where σ^2^ = variance, *I* = mean current, *i* = single-channel current, *N* = number of open channels at peak current, and σ_b_^2^ = background variance. From this equation, γ was calculated by dividing *i* by the driving force (-70 mV; AMPAR reversal potential was ∼0 mV with the recording solutions used). Recordings were discarded if the parabolic fits of the current variance plots had *R*^2^ <0.5 (Traynelis et al., 1993; Hartveit and Veruki, 2007).

### Immunohistochemistry and Image Analysis

Cells were transfected with GFP at plating, as described above. For excitatory synaptic puncta analysis, they were stained for GluA2 and VGLUT1, imaged and analyzed as previously described (Buren et al., 2016). Briefly, they were live stained on DIV 20–22 with a mouse anti-GluA2 primary antibody (Millipore), fixed, and stained with an Alexa-Fluor 568 donkey anti-mouse secondary antibody (Invitrogen), followed by incubation with a guinea pig anti-VGLUT1 primary antibody (Millipore), and then an AMCA-conjugated donkey anti-guinea pig secondary antibody (Jackson ImmunoResearch Laboratories). GFP-positive cells were imaged as a Z-stack using a Zenn Axiovert 200 M fluorescent microscope at 63× magnification with a numerical aperture of 1.4, and flattened within the ZEN 2012 program using the extended depth of focus function. These flattened images were transferred as TIFF files to ImageJ^1^ and split into red, green and blue channels. The background of each channel was removed using the ImageJ subtract background tool, and each channel was manually thresholded. The density of puncta in the red and blue channels, that were at least 40 μm from the cell body and colocalised with a green fluorescent dendritic process connected with the cell being analyzed, was measured using the Analyze Particles tool in ImageJ. Colocalised puncta were defined as contiguous pixels that were above threshold in both the red and blue channels, and were analyzed using the Analyze Particles tool and the colocalization plugin (see footnote 1) in ImageJ.

For spine analysis, cells were fixed on DIV 20–22 by incubation for 20 min with 4% paraformaldehyde (PFA) + 4% sucrose, washing three times with phosphate buffered saline (PBS), and mounted on glass slides (Corning, 2948-75 × 25) using Fluoromount G (SouthernBiotech, 0100-01). Cells were imaged using a Zeiss Axioplan 2 confocal microscope running the ZEN 2009 program, with a single picture being taken of each cell body, as well as three Z stacks, each centered on a segment of a different secondary dendrite which served as the regions of interest (ROIs). These images were exported as TIFFs, and the Z stacks were flattened in ImageJ using the Z project function. Background and noise were removed from the images of the ROIs using the ImageJ subtract background and despeckle functions, respectively. They were then opened in NeuronStudio^2^, and spines along the ROIs were classified using NeuronStudio’s spine classifier tool. The density of each type of spine was calculated for each ROI, and then the densities were averaged across three ROIs per cell to obtain the densities of each spine type for each cell.

### Statistics

All statistics were performed using GraphPad Prism, with one- and two-way ANOVAs and unpaired, two-tailed *t*-tests were performed throughout, as reported in the text. Bonferroni post-tests were applied to test for differences between groups following ANOVAs. All experiments were performed on cells obtained from at least three independent cultures, and “*n*” is the number of cells analyzed. Error bars in all figures represent standard error values of the mean calculated from the number of cells analyzed.

## Results

### HSP Is Impaired in YAC128 Cortical Pyramidal Neurons (CPNs)

**FIGURE 1:**
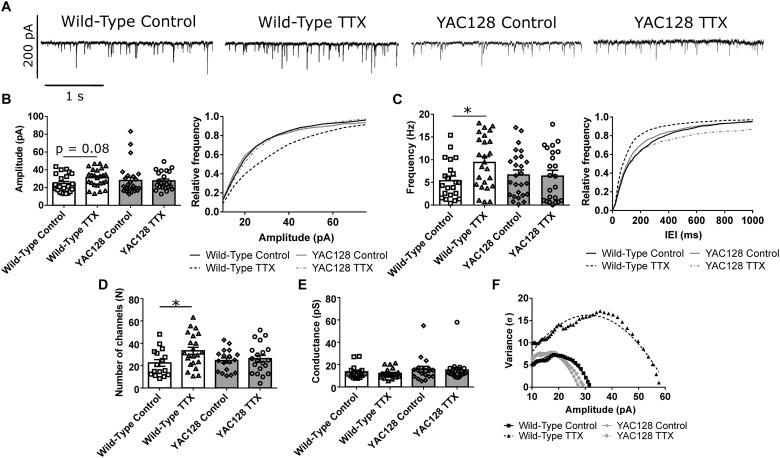
Impaired homeostatic plasticity in YAC128 cortical pyramidal neurons (CPNs). **(A)** Representative traces of miniature excitatory postsynaptic currents (mEPSC) recordings from wild-type (WT) and YAC128 CPNs treated with tetrodotoxin (TTX) or vehicle control. **(B)** mEPSC average amplitude (left panel) and amplitude cumulative probability (right panel). There is a trend toward increased mEPSC amplitude in WT CPNs treated with TTX (*p* = 0.08, Bonferroni *post hoc t*-test), but not in YAC128 CPNs treated with TTX (*p* > 0.99, Bonferroni *post hoc t*-test). **(C)** mEPSC average frequency (left panel) and inter-event interval (IEI) cumulative probability (right panel). There is increased mEPSC frequency in WT CPNs treated with TTX (^∗^*p* < 0.05, Bonferroni *post hoc t*-test), but not in YAC128 CPNs treated with TTX (*p* > 0.99, Bonferroni *post hoc t*-test; TTX *p* = 0.07, genotype/TTX interaction *p* < 0.05, two-way ANOVA). For **(B)** and **(C)**, cell numbers are WT control *n* = 22, WT TTX *n* = 25, YAC128 control *n* = 26, YAC128 TTX *n* = 23; culture batch *N* = 6. **(D)** Number of AMPARs per synapse. WT CPNs treated with TTX had more AMPARs per synapse (^∗^*p* < 0.05, Bonferroni *post hoc t*-test), while YAC128 CPNs treated with TTX did not (*p* > 0.99; TTX *p* < 0.05, two-way ANOVA). **(E)** AMPAR conductance. There was no significant difference in AMPAR conductance between any groups (WT control vs. WT TTX: *p* > 0.99; YAC128 control vs. YAC128 TTX: *p* > 0.99; WT control vs. YAC128 control: *p* = 0.87; WT TTX vs. YAC128 TTX: *p* = 0.50). For **(D)** and **(E)**, cell numbers are WT control *n* = 16, WT TTX *n* = 22, YAC128 control *n* = 18, YAC128 TTX *n* = 20; culture batch *N* = 6. **(F)** Representative current-variance plots for WT and YAC128 CPNs treated with TTX or vehicle control.

To assess the capacity for HSP, cultured CPNs were treated with tetrodotoxin (TTX) or vehicle control (water) for 48 h, after which mEPSCs were recorded (mEPSCs; Figure 1A). mEPSC frequency increased (^∗^*p* < 0.05) and mEPSC amplitude tended to increase (*p* = 0.08) in WT CPNs (Figure 1B,C). However, there was no change in mEPSC frequency or amplitude in YAC128 CPNs (Figure 1B,C), suggesting that YAC128 CPNs are impaired in synaptic upscaling. A non-stationary noise analysis performed on the subset of these traces which also met the standards described above for this analysis (Methods, 2.4) found a significant increase in the number of AMPARs per synapse in WT (^∗^*p* < 0.05) but not YAC128 CPNs treated with TTX (Figure 1D,F). TTX treatment did not alter AMPAR conductance in CPNs of either genotype (Figure 1E,F).

**FIGURE 2:**
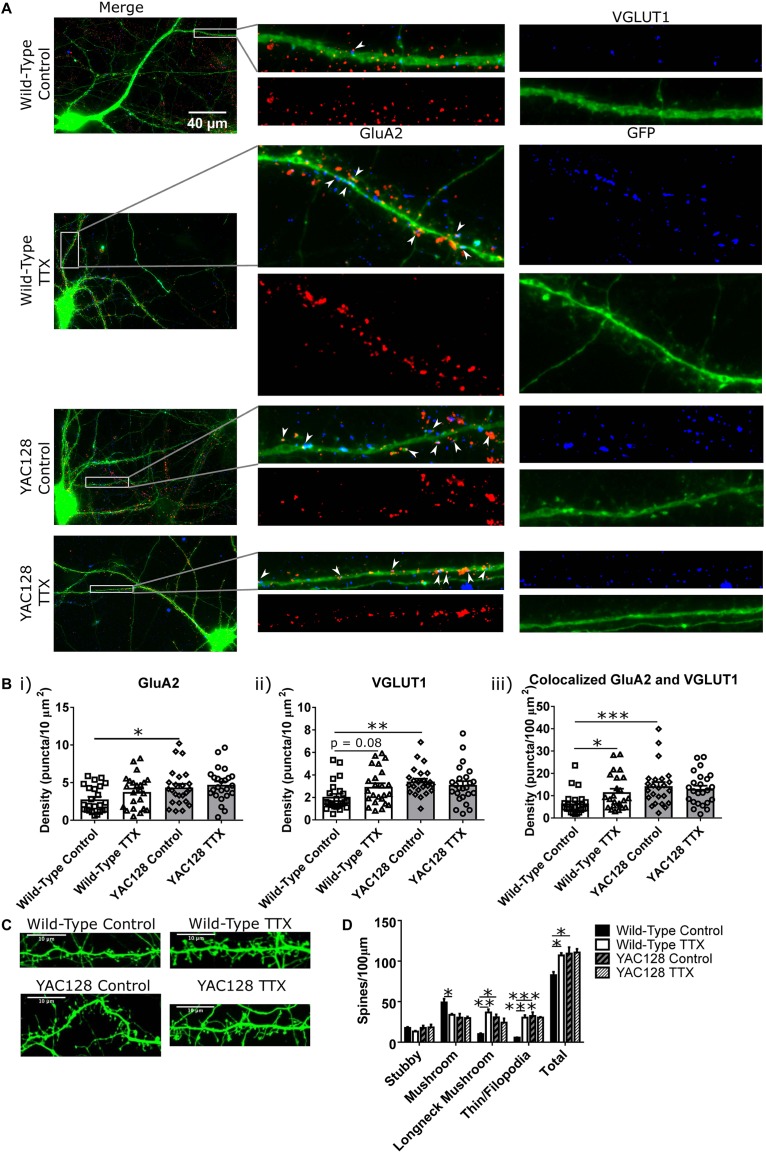
Synaptic changes underlying homeostatic plasticity. **(A)** Representative images of CPNs filled with GFP (green), and stained for GluA2 (red), and VGLUT1 (blue). **(B)** Density of **(i)** GluA2 (genotype *p* < 0.01, two-way ANOVA), **(ii)** VGLUT1 (genotype *p* < 0.05, genotype/TTX interaction *p* < 0.05, two-way ANOVA), and **(iii)** colocalised GluA2 and VGLUT1 puncta (genotype *p* < 0.01, genotype/TTX interaction *p* < 0.05, two-way ANOVA) on CPN dendrites. YAC128 control CPNs had a higher density of all three types of puncta than WT control CPNs (GluA2 ^∗^*p* < 0.05, Bonferroni *post hoc t*-test; VGLUT1 ^∗∗^*p* < 0.01, Bonferroni *post hoc t*-test; colocalised ^∗∗∗^*p* < 0.001, Bonferroni *post hoc t*-test). WT CPNs treated with TTX also showed a tendency to increased VGLUT1 density (*p* = 0.08, Bonferroni *post hoc t*-test) and significantly increased density of colocalised GluA2 and VGLUT1 puncta (^∗^*p* < 0.05, Bonferroni *post hoc t*-test) relative to WT control CPNs. YAC128 CPNs treated with TTX did not differ from either YAC128 control CPNs or WT CPNs treated with TTX (*p* > 0.99, Bonferroni *post hoc t*-test; cells per group *n* = 24, culture batch *N* = 3). **(C)** Representative images of spines on CPNs filled with GFP. **(D)** Spine density, grouped by morphology. WT control CPNs had a higher density of mushroom spines than TTX-treated WT CPNs (^∗^*p* < 0.05, Bonferroni *post hoc t*-test; genotype *p* < 0.05, two-way ANOVA), as well as fewer longneck mushroom spines (mushroom spines with necks > 1 μm in length; ^∗∗^*p* < 0.01, Bonferroni *post hoc t*-test) and filopodia/thin spines (^∗∗∗^*p* < 0.001, Bonferroni *post hoc t*-test). Compared to YAC128 control CPNs, WT control CPNs also had fewer longneck mushroom spines (^∗^*p* < 0.05, Bonferroni *post hoc t*-test; TTX *p* < 0.05, TTX/genotype interaction *p* < 0.01, two-way ANOVA) and filopodia/thin spines (^∗∗∗^*p* < 0.001, Bonferroni *post hoc t*-test; TTX *p* < 0.01, genotype *p* < 0.01, TTX/genotype *p* < 0.01, two-way ANOVA). WT control CPNs also had a lower total spine density than TTX-treated WT CPNs (^∗^*p* < 0.05, Bonferroni *post hoc t*-test) and YAC128 control CPNs (^∗^*p* < 0.05, Bonferroni *post hoc t*-test; TTX *p* < 0.05, genotype *p* < 0.05, two-way ANOVA; culture batches *N* = 3).

Before investigating possible mechanisms of this impairment, we sought to better understand the synaptic changes that accompanied the increased mEPSC amplitude and frequency in WT CPNs, and whether or not these properties were altered in YAC128 CPNs under TTX treatment. Cultured CPNs were transfected with GFP and immunostained for the AMPA receptor subunit GluA2 and the vesicular glutamate transporter VGLUT1 (Figure 2A). GluA2 served as a postsynaptic marker and marker of AMPA receptor (AMPAR) localisation, and VGLUT1 represented a presynaptic marker and marker of glutamate-containing vesicles. Quantification showed that the density of colocalised GluA2 and VGLUT1 puncta was increased (^∗^*p* < 0.05) in WT CPNs treated with TTX relative to those that were not (Figure 2B). These data are consistent with a model in which an increase in synapse density underlies the increase in mEPSC frequency observed in WT CPNs after TTX treatment. Although this would not be considered synaptic scaling, as it involves changing the number of synapses onto a cell rather than the strength of those synapses(Marder and Goaillard, 2006; Watt and Desai, 2010), it is still a form of homeostatic plasticity, as it modifies the properties of cells within a network to preserve the stability of that network (Turrigiano, 2007; Watt and Desai, 2010). Strikingly, there was also a significant increase in density of colocalised puncta (^∗∗∗^*p* < 0.001) and VGLUT1 (^∗∗^*p* < 0.01), as well as GluA2 (^∗^*p* < 0.05), in YAC128 relative to WT CPNs within the control condition (Figure 2B). Given that there is no difference in any of these measures between control and TTX-treated YAC128 CPNs (Figure 2B), this suggests a ceiling effect in which synapse density onto YAC128 CPNs is already increased in response to some other signal before they are exposed to TTX. If so, it is possible that YAC128 CPN synapse density failed to increase in response to TTX because this effect was already saturated.

In addition to modifying the properties of existing synapses, HSP can also occur via addition or elimination of spines and synapses (Tian et al., 2010; Cohen et al., 2011; Mendez et al., 2018). Furthermore, the morphology of spines can also give an indication of their function. For example, mushroom spines are the most stable type of spine, while thin spines are less so (Chidambaram et al., 2019). Given the change in mEPSC frequency observed, spine density and morphology were also examined (Figure 2C,D). WT control CPNs had a higher density of mushroom spines than TTX-treated WT CPNs (^∗^*p* < 0.05). On the other hand, WT control CPNs had a lower density of longneck mushroom spines (defined as mushroom spines with a neck >1 μm long) and filopodia/thin spines than TTX-treated WT CPNs (longneck mushroom: ^∗∗^*p* < 0.01; filopodia/thin: ^∗∗∗^*p* < 0.001) and YAC128 control CPNs (longneck mushroom: ^∗^*p* < 0.05; filopodia/thin: ^∗∗∗^*p* < 0.001). WT control CPNs also had a lower total spine density than TTX-treated WT CPNs (^∗^*p* < 0.05) and YAC128 control CPNs (^∗^*p* < 0.05; Figure 2D).

### BDNF Modulates mEPSC Frequency Increase in Response to Activity Suppression

**FIGURE 3:**
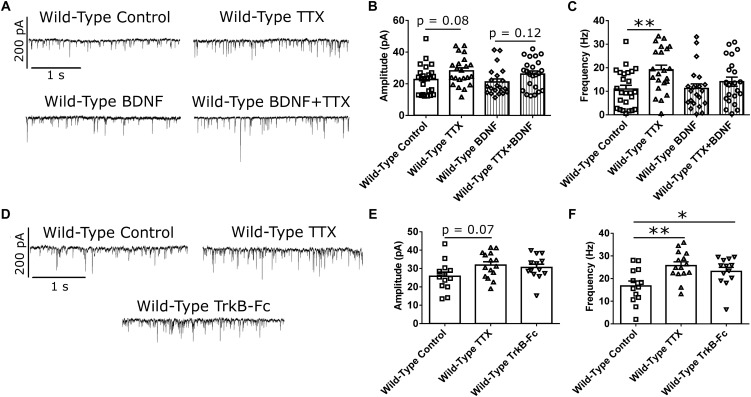
BDNF signaling modulates mEPSC frequency in WT CPNs. **(A)** Representative traces of mEPSC recordings from WT CPNs treated with TTX and/or BDNF. **(B)** mEPSC amplitude of WT CPNs treated with TTX and/or BDNF. By two-way ANOVA, there was no significant *post hoc* difference between any groups, but there was a significant overall effect of TTX (*p* < 0.01); moreover, TTX-treated cells tended to have larger mEPSCs in both vehicle control (*p* = 0.08, Bonferroni *post hoc t*-test) and BDNF-treated (*p* = 0.12, Bonferroni *post hoc t*-test) groups. **(C)** mEPSC frequency of WT CPNs treated with TTX and/or BDNF. Cells treated with only TTX had more frequent mEPSCs than those treated with only vehicle control (^∗∗^*p* < 0.01, Bonferroni *post hoc t*-test; TTX *p* < 0.01, two-way ANOVA). For **(B)** and **(C)**, control *n* = 24, TTX *n* = 22, BDNF *n* = 22, TTX + BDNF = 23, culture batch *N* = 8). **(D)** Representative traces of mEPSC recordings from WT CPNs treated with TTX, TrkB-Fc, or vehicle control. **(E)** mEPSC amplitude of WT CPNs treated with TTX, TrkB-Fc, or vehicle control. TTX-treated cells tended to have larger mEPSCs than those treated with vehicle control (*p* = 0.07, Bonferroni *post hoc t*-test) while TrkB-Fc did not (*p* = 0.24, Bonferroni *post hoc t*-test; *p* = 0.09, one-way ANOVA). **(F)** mEPSC frequency of WT CPNs treated with TTX, TrkB-Fc, or vehicle control. Cells treated with TTX (^∗∗^*p* < 0.01, Bonferroni *post hoc t*-test), and TrkB-Fc (^∗^*p* < 0.05, Bonferroni *post hoc t*-test) both had more frequent mEPSCs than cells treated with vehicle control (*p* < 0.01, one-way ANOVA).

BDNF signaling is thought to be involved in HSP (Rutherford et al., 1998; Turrigiano, 2007; Watt and Desai, 2010). Specifically, BDNF is released in an activity-dependent manner (Poo, 2001), such that suppression of activity in a network of CPNs reduces BDNF release in that network. This reduction in BDNF signaling is thought to be a trigger for synaptic scaling-up (Rutherford et al., 1998; Watt and Desai, 2010). To investigate whether reduced BDNF signaling is required for the changes in mEPSC amplitude and frequency that we observed in our WT cortical cultures at DIV 21 (Rutherford et al., 1998; Watt and Desai, 2010), we first attempted to block homeostatic plasticity in WT CPNs by treating them with BDNF along with TTX. If the effect of TTX is mediated through reduced BDNF signaling, then adding exogenous BDNF at the same time as TTX should block the effect of TTX (Rutherford et al., 1998). Exogenous BDNF did block the increase in mEPSC frequency in response to TTX (Figure 3A,C and Supplementary Figure S1B). However, it did not block the increase in mEPSC amplitude. While there was no significant difference by Bonferroni *post hoc* test between TTX and control cells in either the BDNF or the control group, both groups showed strong trends toward increased mEPSC amplitude in TTX-treated cells (control: *p* = 0.08; BDNF: *p* = 0.12), and there was a significant overall effect of TTX by two-way ANOVA (^∗∗^*p* < 0.01; Figure 3B and Supplementary Figure S1A). To further investigate the role of BDNF in the homeostatic plasticity that we observed, we attempted to reproduce the effect of TTX by scavenging BDNF from our culture medium using TrkB-Fc (Rutherford et al., 1998; Buren et al., 2014). When WT CPNs were treated with TTX or TrkB-Fc for 48 h, both TTX (^∗∗^*p* < 0.01) and TrkB-Fc (^∗^*p* < 0.05) increased mEPSC frequency (Figure 3D,F and Supplementary Figure S1D). TrkB-Fc also had a similar effect as TTX on mEPSC amplitude (Figure 3D,E and Supplementary Figure S1C). Together, these data suggested that, in our DIV 21 WT cortical cultures, reduced BDNF signaling is a trigger for HSP in response to activity suppression.

### Pridopidine Restores Homeostatic Plasticity in YAC128 CPNs Through Stimulation of the Sigma-1 Receptor

We hypothesized that the homeostatic plasticity deficit observed in YAC128 CPNs is a result of the disruption of a signaling pathway(s) involved in this process that is also known to be impaired in HD. This disruption leads CPNs to undergo a homeostatic increase in synapse density under baseline conditions and prevents activity blockade from inducing further HSP. Specifically, since BDNF signaling is impaired in HD (Zuccato and Cattaneo, 2007), we speculate that the level of BDNF signaling in YAC128 CPNs is low enough under baseline conditions to prevent any further reduction from engaging HSP pathways. It is also possible that reduced BDNF signaling engages these pathways under baseline conditions in YAC128 CPNs, leading to the increased synapse and spine density we showed in Figure 2. In addition to BDNF, another key process involved in HSP is regulation of somatic calcium levels (Watt and Desai, 2010; Turrigiano, 2011). In this regard, the drug pridopidine, which has shown promise for the treatment of HD motor symptoms in clinical trials (Kieburtz et al., 2018) is of interest. Recent research has demonstrated that pridopidine boosts the BDNF signaling pathway and is involved in stabilizing ER calcium signaling (Geva et al., 2016; Ryskamp et al., 2017). Therefore, we hypothesized that pridopidine might restore homeostatic plasticity in response to suppression of activity in YAC128 CPNs by normalizing BDNF and/or calcium signaling under baseline conditions.

**FIGURE 4:**
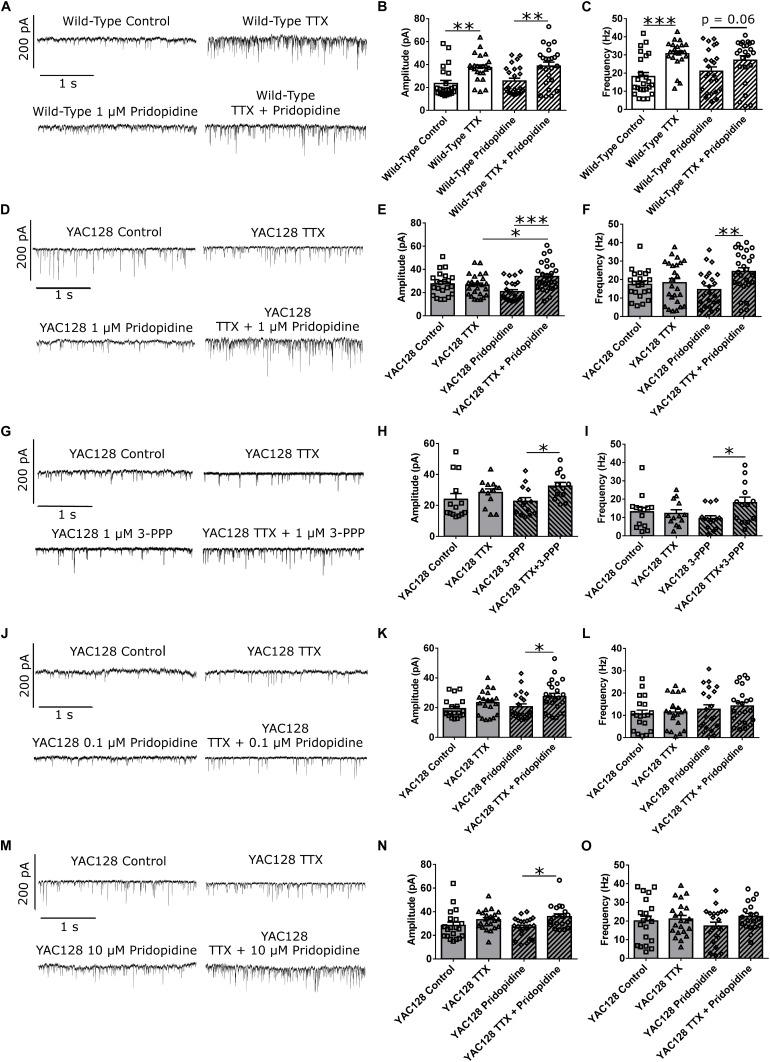
Pridopidine and S1R agonist 3-PPP both restore homeostatic plasticity at a 1 μM concentration. **(A)** Representative traces of mEPSC recordings from WT CPNs treated with TTX and/or 1 μM pridopidine. **(B)** mEPSC amplitude of WT CPNs treated with TTX and/or 1 μM pridopidine. Cells treated with only TTX had larger mEPSCs than those treated with only vehicle control (^∗∗^*p* < 0.01, Bonferroni *post hoc t*-test), and cells treated with TTX and pridopidine had larger mEPSCs than those treated with only pridopidine (^∗∗^*p* < 0.01, Bonferroni *post hoc t*-test; TTX *p* < 0.001, two-way ANOVA). **(C)** mEPSC frequency of WT CPNs treated with TTX and/or 1 μM pridopidine. Cells treated with only TTX had more frequency mEPSCs than those treated with only vehicle control (^∗∗∗^*p* < 0.001, Bonferroni *post hoc t*-test), while those treated with both TTX and pridopidine showed a non-significant trend toward more frequent mEPSCs than those treated with only pridopidine (*p* = 0.06, Bonferroni *post hoc t*-test; TTX *p* < 0.001, two-way ANOVA). For both **(B)** and **(C)**, cells per group *n* = 24, culture batch *N* = 4. **(D)** Representative traces of mEPSC recordings from YAC128 CPNs treated with TTX and/or 1 μM pridopidine. **(E)** mEPSC amplitude of YAC128 CPNs treated with TTX and/or 1 μM pridopidine. Cells treated with both TTX and pridopidine had higher amplitude mEPSCs than those treated with only TTX (^∗^*p* < 0.05, Bonferroni *post hoc t*-test), or pridopidine (^∗∗∗^*p* < 0.001, Bonferroni *post hoc t*-test; TTX *p* < 0.01, TTX/pridopidine interaction *p* < 0.001, two-way ANOVA). **(F)** mEPSC frequency of YAC128 CPNs treated with TTX and/or 1 μM pridopidine. Cells treated with both TTX and pridopidine had more frequent mEPSCs than those treated with pridopidine alone (^∗∗^*p* < 0.01, Bonferroni *post hoc t*-test; TTX *p* < 0.05, TTX/pridopidine interaction *p* < 0.05, two-way ANOVA). For **(E)** and **(F)**, control *n* = 22, TTX *n* = 25, pridopidine *n* = 24, TTX + pridopidine *n* = 27, culture batch *N* = 4. **(G)** Representative traces of mEPSC recordings from YAC128 CPNs treated with TTX and/or 3-PPP. **(H)** mEPSC amplitude from YAC128 CPNs treated with TTX and/or 3-PPP. Cells treated with both 3-PPP and TTX had larger mEPSCs than those treated with only 3-PPP (^∗^*p* < 0.05, Bonferroni *post hoc t*-test; TTX *p* < 0.05, two-way ANOVA). **(I)** mEPSC frequency from YAC128 CPNs treated with TTX and/or 3-PPP. Cells treated with both 3-PPP and TTX had more frequent mEPSCs than those treated with only 3-PPP (^∗^*p* < 0.05, Bonferroni *post hoc t*-test; TTX/3-PPP interaction *p* < 0.05, TTX *p* = 0.09, two-way ANOVA).; For **(H)** and **(I)**, control *n* = 14, TTX *n* = 13, 3-PPP *n* = 14, TTX/3-PPP *n* = 13, culture batch *N* = 12. **(J)** Representative traces of mEPSC recordings from YAC128 CPNs treated with TTX and/or 100 nM pridopidine. **(K)** mEPSC amplitude of YAC128 CPNs treated with TTX and/or 100 nM pridopidine. YAC128 CPNs treated with both TTX and pridopidine had higher mEPSC amplitude than those treated with only pridopidine (^∗^*p* < 0.05, Bonferroni *post hoc t*-test; TTX *p* < 0.01, two-way ANOVA). **(L)** mEPSC frequency of YAC128 CPNs treated with TTX and/or 100 nM pridopidine. There was no difference in mEPSC frequency between any groups (control vs. TTX: *p* > 0.99; pridopidine vs. pridopidine+TTX: *p* > 0.99; control vs. pridopidine: *p* = 0.83; TTX vs. pridopidine+TTX: *p* = 0.56, Bonferroni *post hoc t*-test). For **(K)** and **(L)**, control *n* = 19, TTX *n* = 20, pridopidine *n* = 21, TTX + pridopidine *n* = 23, culture batch *N* = 4. **(M)** Representative traces of mEPSC recordings from YAC128 CPNs treated with TTX and/or 10 μM pridopidine. **(N)** mEPSC amplitude of YAC128 CPNs treated with TTX and/or 10 μM pridopidine. Cells treated with both TTX and pridopidine had larger mEPSCs than those treated with only pridopidine (^∗^*p* < 0.05, Bonferroni *post hoc t*-test; TTX *p* < 0.01, two-way ANOVA). **(O)** mEPSC frequency of YAC128 CPNs treated with TTX and/or 10 μM pridopidine. There was no difference in frequency between any groups (control vs. TTX: *p* = 0.92; pridopidine vs. pridopidine+TTX: *p* = 0.18; control vs. pridopidine: *p* = 0.72; TTX vs. pridopidine+TTX: *p* > 0.99, Bonferroni *post hoc t*-test). For **(N)** and **(O)**, control *n* = 20, TTX *n* = 22, pridopidine *n* = 21, TTX + pridopidine = 19; culture batch *N* = 4.

We treated WT CPNs with 1 μM pridopidine. Given that pridopidine upregulates BDNF signaling (Geva et al., 2016) and that exogenous BDNF at least partially blocked HSP (Figure 3A–C), pridopidine might be expected to have a blunting effect on homeostatic plasticity in WT CPNs, which have a higher baseline level of BDNF signaling than do YAC128 CPNs. These cells showed increased mEPSC amplitude in response to TTX regardless of whether they were (^∗∗^*p* < 0.01) or were not (^∗∗^*p* < 0.01) treated with pridopidine (Figure 4A,B and Supplementary Figure S2A). However, whereas cells treated with only TTX had increased mean mEPSC frequency relative to those treated with vehicle control (^∗∗∗^*p* < 0.001), the difference in mean mEPSC frequency between cells treated with both TTX and pridopidine or with pridopidine alone did not reach statistical significance (*p* = 0.06), suggesting an attenuated effect of TTX on mEPSC frequency (Figure 4C and Supplementary Figure S2B). These data are consistent with a model in which 1 μM pridopidine impaired HSP in response to TTX in WT CPNs.

To test the effect of pridopidine on HSP in YAC128 CPNs, cultured CPNs were treated with pridopidine (0.1, 1, or 10 μM) or vehicle control (water) for 48 h before TTX or vehicle control was also added to the culture medium. After treatment with TTX, mEPSCs were recorded from these neurons. In cultures treated with either the 0.1 or 10 μM pridopidine concentration, YAC128 CPNs showed increased mEPSC amplitude in response to TTX (^∗^*p* < 0.05), but mean mEPSC frequency remained unchanged (Figure 4J–O and Supplementary Figures S3C–F). In contrast, YAC128 CPNs treated with 1 μM pridopidine showed increases in both mean mEPSC amplitude (^∗∗∗^*p* < 0.001) and frequency (^∗∗^*p* < 0.01; Figure 4D–F and Supplementary Figures S3A,B) in response to TTX. Consistent with our previous results, cells treated with vehicle control instead of pridopidine did not show increased mEPSC amplitude or frequency in response to TTX. These results indicate that pridopidine restores homeostatic plasticity in response to activity suppression in YAC128 CPNs. This restoration occurs in a dose-dependent manner, with maximal effect at 1 μM and decreasing efficacy at higher and lower doses.

Pridopidine is an agonist of the sigma-1 receptor (S1R) (Geva et al., 2016; Ryskamp et al., 2017), which, among other functions, regulates both BDNF signaling (Kikuchi-Utsumi and Nakaki, 2008; Fujimoto et al., 2012) and calcium signaling between the mitochondria and ER (Hayashi and Su, 2007; Ryskamp et al., 2017), which could influence homeostatic plasticity (Marder and Goaillard, 2006; Turrigiano, 2007; Watt and Desai, 2010). With this in mind, we attempted to restore TTX-induced homeostatic plasticity in YAC128 CPNs by treating cultures with 3-PPP, another S1R agonist (Ryskamp et al., 2017). Cells that were treated with 1 μM 3-PPP and TTX showed higher mean mEPSC amplitude (^∗^*p* < 0.05) and frequency (^∗^*p* < 0.05) than those treated with only 3-PPP (Figure 4G–I and Supplementary Figure S4). There was no difference in mean mEPSC amplitude or frequency between TTX-treated and control cells that were not treated with 3-PPP. This suggests that S1R agonism contributes to pridopidine’s restoration of homeostatic plasticity in YAC128 CPNs.

**FIGURE 5:**
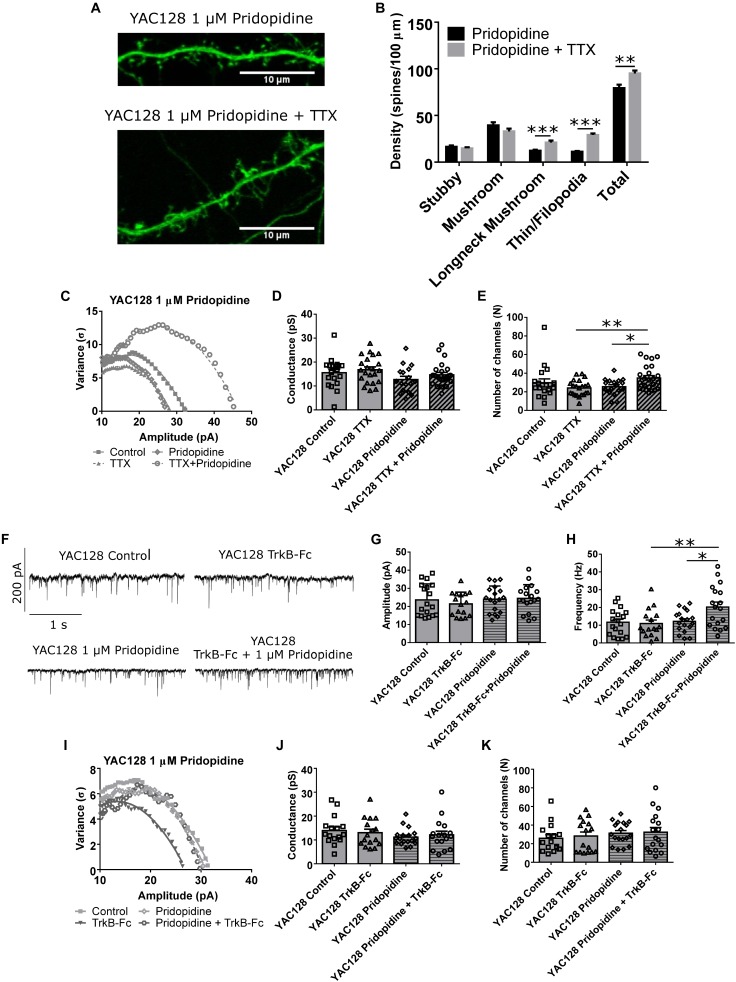
Pridopidine restores other aspects of homeostatic plasticity. **(A)** Representative images of spines on CPNs filled with GFP. **(B)** Spine density of YAC128 CPNs treated with 1 μM pridopidine and TTX or vehicle control. Cells treated with TTX and pridopidine had a higher density of longneck mushroom spines (^∗∗∗^*p* < 0.001, unpaired *t*-test) and filopodia/thin spines (^∗∗∗^*p* < 0.001, unpaired *t*-test), as well as a higher total spine density (^∗∗^*p* < 0.01, unpaired *t*-test) than those treated with only pridopidine (cells per group *n* = 18, culture batches *N* = 3) **(C)** Representative current-variance plots from YAC128 CPNs treated with TTX and/or 1 μM pridopidine. **(D)** Peak conductance of synapses onto YAC128 CPNs treated with TTX and/or 1 μM pridopidine, as determined by non-stationary noise analysis. There was no significant difference between any groups (control vs. TTX: *p* = 0.95; pridopidine vs. pridopidine+TTX: *p* = 0.51; control vs. pridopidine: *p* = 0.25; TTX vs. pridopidine+TTX: *p* = 0.40, Bonferroni *post hoc t*-test), although there was a significant effect of pridopidine on conductance (pridopidine *p* < 0.05, two-way ANOVA). **(E)** Number of AMPARs per synapse onto YAC128 CPNs treated with TTX and/or 1 μM pridopidine, as determined by non-stationary noise analysis. There were more AMPARs in cells treated with both TTX and pridopidine than in cells treated with only TTX (^∗∗^*p* < 0.01, Bonferroni *post hoc t*-test) or pridopidine (^∗^*p* < 0.05, Bonferroni *post hoc t*-test; TTX/pridopidine interaction *p* < 0.01, two-way ANOVA). For **(D)** and **(E)**, control *n* = 19, TTX *n* = 20, pridopidine *n* = 21, TTX + pridopidine *n* = 23, culture batch *N* = 4. **(F)** Representative traces of mEPSC recordings from YAC128 CPNs treated with TrkB-Fc and/or pridopidine. **(G)** mEPSC amplitude of YAC128 CPNs treated with TrkB-Fc and/or pridopidine. There was no significant difference between groups (control vs. TrkB-Fc: *p* = 0.81; pridopidine vs. TrkB-Fc: *p* > 0.99; control vs. pridopidine: *p* > 0.99; TrkB-Fc vs. pridopidine+TrkB-Fc: *p* = 0.49, Bonferroni *post hoc t*-test). **(H)** mEPSC frequency of YAC128 CPNs treated with TrkB-Fc and/or pridopidine. Cells treated with TrkB-Fc and pridopidine had more frequent mEPSCs than both those treated with only TrkB-Fc (^∗∗^*p* < 0.01, Bonferroni *post hoc t*-test) or only pridopidine (^∗^*p* < 0.05, Bonferroni *post hoc t*-test; pridopidine *p* < 0.05, TrkB-Fc/pridopidine interaction *p* < 0.05, two-way ANOVA). **(I)** Representative current-variance plots from YAC128 CPNs treated with TrkB-Fc and/or pridopidine. **(J)** Peak conductance of synapses onto YAC128 CPNs treated with TrkB-Fc and/or pridopidine, as determined by non-stationary noise analysis. There was no significant difference between any groups (control vs. TrkB-Fc: *p* > 0.99; pridopidine vs. pridopidine+TrkB-Fc: *p* > 0.99; control vs. pridopidine: *p* = 0.31; TrkB-Fc vs. pridopidine+TrkB-Fc: *p* > 0.99). **(K)** Number of AMPARs per synapse onto YAC128 CPNs treated with TrkB-Fc and/or pridopidine, as determined by non-stationary noise analysis. There was no significant difference between any groups (control vs. TrkB-Fc: *p* > 0.99; pridopidine vs. pridopidine+TrkB-Fc: *p* > 0.99; control vs. pridopidine: *p* = 0.68; TrkB-Fc vs. pridopidine+TrkB-Fc: *p* = 0.97). For **(G)**, **(H)**, **(J)**, and **(K)**, control *n* = 18, TrkB-Fc *n* = 16, pridopidine *n* = 18, TrkB-Fc + pridopidine *n* = 18; culture batch *N* = 6.

Other aspects of the observed homeostatic plasticity were also analyzed. Spine density and morphology showed increased density of both longneck mushroom (^∗∗∗^*p* < 0.001) and filopodia/thin spines (^∗∗∗^*p* < 0.001), as well as total spine density (^∗∗^*p* < 0.01; Figure 5A,B) in TTX-treated YAC128 CPNs that had first been incubated for 48 h with 1 μM pridopidine. Non-stationary noise analysis also revealed that at the 1 μM dose, cells treated with both pridopidine and TTX had more AMPARs at each synapse than cells treated with only TTX (^∗∗^*p* < 0.01) or only pridopidine (^∗^*p* < 0.05, Figure 5C–E), suggesting that pridopidine affects AMPAR trafficking pathways typically associated with synaptic upscaling (Rutherford et al., 1998; Turrigiano et al., 1998; Marder and Goaillard, 2006; Turrigiano, 2007; Watt and Desai, 2010). In addition, we tested whether YAC128 CPNs’ response to BDNF scavenging was also impaired, as would be expected if BDNF signaling is reduced in these cells under baseline conditions, and whether pridopidine could restore this. We treated YAC128 CPNs with 1 μM pridopidine for 48 h, and then with TrkB-Fc (together with pridopidine) for 48 h. Cells treated with both pridopidine and TrkB-Fc had higher mean mEPSC frequency than cells treated with only TrkB-Fc (^∗∗^*p* < 0.01) or pridopidine (^∗^*p* < 0.05; Figure 5F,H and Supplementary Figure S5), although there was no significant difference in mean mEPSC amplitude between any groups (Figure 5G). There was also no difference in number of AMPARs per synapse or the peak conductance of those receptors, as determined by non-stationary noise analysis (Figure 5I–K). This suggests that the effect of pridopidine on TTX-induced increase in mEPSC frequency is mediated by its effect on the BDNF signaling pathway.

## Discussion

We investigated functional synaptic responses to a homeostatic plasticity stimulus in cortical neurons from a mouse model of HD. After 48 h of TTX treatment, cultured CPNs from WT FVB/N mice showed increased mEPSC frequency and a strong trend, which was significant in some sets of experiments, toward increased mEPSC amplitude. The neurons also exhibited increased synapse and spine density, and number of AMPARs per synapse. YAC128 CPNs, in contrast, did not change in any of these measures, suggesting a deficit in homeostatic plasticity. BDNF was involved in the observed effect of TTX on mEPSC frequency in WT CPNs. We were able to restore TTX treatment-induced homeostatic plasticity in YAC128 CPNs with pridopidine, a drug which improved motor deficits in HD clinical trials and significantly enhances BDNF signaling in rodents (Geva et al., 2016; Kieburtz et al., 2018). Our quantification show that pridopidine restored homeostatic plasticity in a dose-dependent manner, with maximum effect at 1 μM. Consistent with pridopidine’s proposed mechanism of action (Geva et al., 2016; Ryskamp et al., 2017), homeostatic plasticity was also restored by the S1R agonist 3-PPP.

### TTX Induced Multiple Forms of Homeostatic Plasticity

After 48 h of treatment with TTX we expected to see synaptic scaling. This is a form of homeostatic plasticity in which the strength of all of the synapses onto a neuron increases or decreases in response to changes in the neuron’s level of activity. In excitatory neurons such as CPNs, a prolonged decrease in activity results in increased synaptic strength (Rutherford et al., 1998; Marder and Goaillard, 2006; Turrigiano, 2007; Watt and Desai, 2010). This is usually assessed by measuring the amplitude and frequency of mEPSCs, small inward currents produced by the stochastic release of individual glutamate-containing synaptic vesicles and mediated by AMPAR activation. Increased mEPSC amplitude is typically interpreted as reflecting postsynaptic changes, while increased frequency is considered to reflect presynaptic changes. Neurons undergoing synaptic scaling at or before DIV 14 typically exhibit only postsynaptic changes. However, between DIV 14 and 18 they undergo a shift toward mixed pre- and postsynaptic scaling (Wierenga et al., 2006). Consistent with this, the WT cells used in our study, which were recorded at DIV 21, showed increases in both mEPSC amplitude and frequency, with frequency changes occurring more consistently. Non-stationary noise analysis indicated that WT CPNs had an increased number of AMPARs per synapse after treatment with TTX, a typical mechanism of mEPSC synaptic up-scaling. These same cells also showed increased spine and synapse density, consistent with the observed increase in mEPSC frequency. While not consistent with synaptic scaling, this has been documented as another form of homeostatic plasticity (Kirov et al., 1999; Turrigiano, 2007). The morphology of spines on TTX-treated WT CPNs was also shifted toward types which appeared less mature and suggested the formation of novel synapses (Kirov et al., 1999). Thus, at DIV 20–22 in our culture system WT CPNs treated with TTX for 48 h increase both AMPAR number per synapse and synapse density.

### Impairment of Synapse Density Change in Response to TTX in YAC128 CPNs Is Mediated by Altered BDNF Signaling

While WT CPNs underwent homeostatic plasticity in response to activity suppression by TTX treatment, YAC128 CPNs’ mEPSCs did not change in amplitude or frequency. Further examination showed that both control and TTX-treated YAC128 CPNs exhibited synapse and spine density, and spine morphology, more similar to that of TTX-treated than control WT CPNs. This suggests that a homeostatic increase in synapse density is occluded in YAC128 CPNs.

BDNF signaling is impaired in HD (Zuccato and Cattaneo, 2007; Plotkin and Surmeier, 2015) and has been reported to play a critical role in HSP. BDNF is released from CPNs in an activity-dependent manner, and reduced BDNF is thought to be a trigger for synaptic up-scaling after activity suppression (Rutherford et al., 1998; Turrigiano, 2007; Watt and Desai, 2010). Our data in WT CPNs, showing that scavenging BDNF from the medium with TrkB-Fc over 48 h mimics the effect of TTX, and that exogenous BDNF in the medium prevents the TTX-induced increase in mEPSC frequency, support this model. Thus, we hypothesized that a baseline deficit in BDNF signaling in YAC128 CPNs renders them insensitive to further reductions in BDNF, thereby occluding TTX-induced homeostatic plasticity. Moreover, reduced BDNF signaling may underlie a homeostatic increase in synaptogenesis during development in culture, resulting in the elevated density of immature-appearing spines and synapses on DIV 20–22 YAC128 CPNs, and preventing further modulation of synapse density following TTX. Supporting this idea, we found that YAC128 CPNs treated with 1 μM pridopidine, a drug previously shown to upregulate BDNF signaling (Geva et al., 2016), exhibited a similar synapse morphology and density as control WT CPNs, and also responded to 48 h TTX treatment with an increase in synapses as found in TTX-treated WT CPNs. It is interesting that YAC128 CPNs pre-treated with 1 μM pridopidine showed increased mEPSC frequency but not amplitude following BDNF scavenging, suggesting that modulation of BDNF signaling plays a larger role in regulating synapse numbers than AMPAR content. Together, these data suggest that reduced BDNF signaling in HD impairs a homeostatic increase in synapse number, but not strengthening of those synapses. Synaptic up-scaling by insertion of AMPARs appears to be largely mediated by some other pathway, which is also impaired in HD and restored by pridopidine.

### Pridopidine Restores Homeostatic Plasticity in YAC128 CPNs by Stimulating the Sigma-1 Receptor

Pridopidine is a drug which has been investigated as a possible treatment for HD (Kieburtz et al., 2018). It was initially identified as a dopamine stabilizer (Dyhring et al., 2010), but recent studies suggest that its effect on HD is largely mediated through the S1R (Sahlholm et al., 2013, 2015). Stimulation of S1R by pridopidine normalizes multiple biochemical pathways which are disrupted in HD, including normalization of BDNF signaling and calcium homeostasis (Geva et al., 2016; Ryskamp et al., 2017). Furthermore, while the mechanisms by which pridopidine normalizes these pathways is still under study, recent data suggest that its agonism of S1R enhances BDNF axonal transport (Ionescu et al., 2019) and elevates BDNF transcripts (Geva, unpublished results). Thus, pridopidine treatment should not only increase the level of BDNF signaling in YAC128 CPNs, but should also restore their ability to dynamically modulate BDNF release. As these pathways in particular are involved in homeostatic plasticity, this suggested that pridopidine might restore homeostatic plasticity.

The pridopidine concentrations used in these experiments were selected based on the known binding affinity to the S1R, which we hypothesized would be the receptor driving the effects in our experimental system. The EC50 of pridopidine to the S1R is ∼ 100 nM as determined by *in vitro* binding assays (Johnston et al., 2018). In previous experiments we demonstrated various effects which were mediated by the S1R, and potent at the range of 100 nM–1 μM. These effects include upregulation of the BDNF pathway (Geva et al., 2016), rescue of spines and calcium homeostasis (Ryskamp et al., 2017, 2019), and enhancement of axonal BDNF transport (Ionescu et al., 2019). In addition, these concentrations correlate to the drug brain exposure levels of pridopidine used in clinical trials (Geva, unpublished data). Specifically, these concentrations show a very selective target engagement of the S1R *in vivo* (Geva et al., 2016; Ryskamp et al., 2017; Ionescu et al., 2019). In a recently completed phase 2 trial in HD patients, the low dose (45 mg twice a day), which shows selective S1R brain occupancy, showed significant slowing of functional decline, while the higher doses were less effective (Reilmann et al., 2018).

We found that pre-treatment with pridopidine restored homeostatic plasticity in YAC128 CPNs in a concentration-dependent manner. While all tested pridopidine concentrations (0.1, 1, and 10 μM) restored the mean mEPSC amplitude increase in response to TTX treatment, only pre-treatment with 1 μM pridopidine was able to restore the increase in mean mEPSC frequency. Furthermore, in WT CPNs, pridopidine pre-treatment did not impact the mean mEPSC amplitude increase in response to 48 h TTX exposure, but it did attenuate the increase in mean mEPSC frequency after activity deprivation. Taken together, these data suggest that the mEPSC frequency response to 48 h TTX is more sensitive to BDNF levels than the amplitude response. As previous work showed BDNF signaling is reduced in HD (Zuccato and Cattaneo, 2007; Plotkin and Surmeier, 2015), it is possible that baseline BDNF signaling must be within a certain range in order for its reduction in response to 48 h TTX treatment to increase synapse density. If so, then its level may be too low in YAC128 CPNs, but elevated to a healthy range by 1 μM pridopidine; further, 10 μM pridopidine may increase BDNF in YAC128 CPNs to a level no longer within this range. 1 μM pridopidine may achieve the same effect in WT CPNs. Alternatively, high concentrations of pridopidine may engage other pathways which inhibit the homeostatic modulation of synapse density, differentially in WT and YAC128 CPNs. Further experiments are required to test these possibilities.

This leaves the question of how pridopidine restores the effect of TTX treatment on synaptic AMPAR content. BDNF signaling is only one of the pathways that pridopidine normalizes through stimulation of the S1R (Geva et al., 2016). We found that the S1R agonist 3-PPP also restored both mEPSC amplitude and frequency increases in response to TTX. This suggests that another process downstream of the S1R, in addition to BDNF, is involved in the restoration of homeostatic plasticity. Of interest to us is calcium homeostasis, which is also restored by pridopidine (Ryskamp et al., 2017). Calcium signaling is involved in homeostatic plasticity through a number of pathways, including CamKIV (Ibata et al., 2008) and Homer1a (Diering et al., 2017); the latter pathway is involved in HSP via regulating ER calcium release. Calcium homeostasis is disrupted in HD through mechanisms such as sensitisation of the IP3R, affecting ER calcium release (Tang et al., 2003), and altered NMDAR localisation and subunit composition (Milnerwood and Raymond, 2010; Milnerwood et al., 2010, 2012). S1R agonists have been found to modulate both of these pathways (Liang and Wang, 1998; Ryskamp et al., 2017). We consider calcium homeostasis to be the most likely pathway by which pridopidine restores the synaptic up-scaling (amplitude) response to TTX.

### Homeostatic Plasticity, S1R, and HD

While most HD research has focused on the striatum, which is the first brain structure in which cell death occurs (MacDonald et al., 1993), the cortex is known to play an important role in the disease’s early cognitive and psychiatric symptoms (Thu et al., 2010; Estrada-Sánchez et al., 2015). The deficit in CPN homeostatic plasticity reported here could contribute to these symptoms. Drugs like pridopidine, which normalize this process, could improve these symptoms.

Another neurodegenerative disease in which homeostatic plasticity has been implicated is Alzheimer’s disease (AD) (Small, 2008). HSP in healthy cells to compensate for synaptic dysfunction in unhealthy cells is hypothesized to contribute both to cognitive dysfunction in AD and spread of neuronal dysfunction to other brain areas. Aberrant cortical HSP in HD could also, in theory, contribute to pathology in brain areas outside of the cortex. The deficit which we documented has the potential to decrease the overall level of activity of affected cortical areas (Watt and Desai, 2010). Such a decrease would lead to reduced activity-dependent release of BDNF by CPNs onto striatal SPNs, compounding the BDNF secretion deficit mediated by reduced expression and axonal transport (Zuccato and Cattaneo, 2007; Virlogeux et al., 2018), and contributing to decreased neurotrophic support of SPNs, which has been proposed to cause the death of these cells through ‘withering’ (Plotkin and Surmeier, 2015).

Homeostatic plasticity has also been investigated in sleep. Homer1a-dependent synaptic down-scaling, reduction of the strength of all of the synapses onto a cell, has been reported during sleep (Diering et al., 2017). Factors involved in up- and down-scaling do not overlap greatly (Turrigiano, 2011), so our finding of impairment in TTX-induced homeostatic plasticity does not necessarily imply that down-scaling is also impaired in HD. However, evidence from clinical neurostimulation does support this idea (Calabresi et al., 2016). Further, Homer1a is part of the BDNF signaling pathway, and its expression is decreased in the Q175 HD mouse model, and restored by pridopidine (Geva et al., 2016).

Finally, our findings add to growing evidence for S1R as a potential pharmacological target for the treatment of HD and other neurodegenerative disorders. S1R agonists have been found to improve symptoms of depression and anxiety, which are among the most common psychiatric symptoms of HD (Berrios et al., 2001). S1R is a relatively recently discovered receptor, but it has already been associated with many pathways implicated in neurodegenerative diseases, including calcium homeostasis, neurotrophic support and synaptic transmission (Kourrich et al., 2012; Geva et al., 2016; Ryskamp et al., 2017). Together with our results, these studies suggest that S1R is a promising pharmacological target for HD, as well as for other neurodegenerative disorders.

## Author Contributions

AS-D was involved in the design of all the experiments included in this manuscript, acquired and analyzed the data for the majority of those experiments, and wrote the majority of this manuscript. WN acquired and analyzed the data for experiments included in this manuscript, and contributed to writing the section “Materials and Methods”. LZ acquired and analyzed the data for experiments included in this manuscript. MG and MH contributed to the design of experiments included in this manuscript. LR conceived the research questions, contributed to the design of experiments, supervised the data analysis, and contributed to the manuscript writing and revision. All authors reviewed and approved the manuscript.

## Conflict of Interest Statement

MG and MH were employed by Teva Pharmaceutical Industries Ltd. This company also provided financial and material support to LR in the form of research funding, and the drug pridopidine, used to support some of the experiments conducted in this study. The remaining authors declare that the research was conducted in the absence of any commercial or financial relationships that could be construed as a potential conflict of interest.

## Footnotes

1 https://imagej.nih.gov/ij/plugins/colocalization.html

2 http://research.mssm.edu/cnic/tools-ns.html

## References

[B1] Berrios G. E. Wagle A. C. Marková I. S. Wagle S. A. Ho L. W. Rubinsztein D. C. (2001). Psychiatric symptoms and CAG repeats in neurologically asymptomatic Huntington’s disease gene carriers. *Psychiatry Res.* 102 217–225. 10.1016/S0165-1781(01)00257-8 11440772

[B2] Buren C. Parsons M. P. Smith-Dijak A. Raymond L. A. (2016). Impaired development of cortico-striatal synaptic connectivity in a cell culture model of Huntington’s disease. *Neurobiol. Dis.* 87 80–90. 10.1016/j.nbd.2015.12.009 26711622

[B3] Buren C. Wang L. Smith-Dijak A. Raymond L. A. (2014). Region-specific pro-survival signaling and global neuronal protection by wild-type huntingtin. *J. Huntingt. Dis.* 3 365–376. 10.3233/JHD-140122 25575958

[B4] Calabresi P. Pisani A. Rothwell J. Ghiglieri V. Obeso J. A. Picconi B. (2016). Hyperkinetic disorders and loss of synaptic downscaling. *Nat. Neurosci.* 19 868–875. 10.1038/nn.4306 27351172

[B5] Cepeda C. Wu N. André V. M. Cummings D. M. Levine M. S. (2007). The corticostriatal pathway in Huntington’s disease. *Prog. Neurobiol.* 81 253–271. 10.1016/j.pneurobio.2006.11.001 17169479 PMC1913635

[B6] Chidambaram S. B. Rathipriya A. G. Bolla S. R. Bhat A. Ray B. Mahalakshmi A. M. (2019). Dendritic spines: revisiting the physiological role. *Prog. Neuropsychopharmacol. Biol. Psychiatry* 92 161–193. 10.1016/j.pnpbp.2019.01.005 30654089

[B7] Cohen J. E. Lee P. R. Chen S. Li W. Fields R. D. (2011). MicroRNA regulation of homeostatic synaptic plasticity. *Proc. Natl. Acad. Sci. U.S.A.* 108 11650–11655. 10.1073/pnas.1017576108 21697510 PMC3136313

[B8] Diering G. H. Nirujogi R. S. Roth R. H. Worley P. F. Pandey A. Huganir R. L. (2017). Homer1a drives homeostatic scaling-down of excitatory synapses during sleep. *Science* 355 511–515. 10.1126/science.aai8355 28154077 PMC5382711

[B9] Dyhring T. Nielsen E. Ø. Sonesson C. Pettersson F. Karlsson J. Svensson P. (2010). The dopaminergic stabilizers pridopidine (ACR16) and (-)-OSU6162 display dopamine D2 receptor antagonism and fast receptor dissociation properties. *Eur. J. Pharmacol.* 628 19–26. 10.1016/j.ejphar.2009.11.025 19919834

[B10] Estrada-Sánchez A. M. Burroughs C. L. Cavaliere S. Barton S. J. Chen S. Yang X. W. (2015). Cortical efferents lacking mutant huntingtin improve striatal neuronal activity and behavior in a conditional mouse model of huntington’s disease. *J. Neurosci.* 35 4440–4451. 10.1523/JNEUROSCI.2812-14.2015 25762686 PMC4355206

[B11] Fujimoto M. Hayashi T. Urfer R. Mita S. Su T.-P. (2012). Sigma-1 receptor chaperones regulate the secretion of brain-derived neurotrophic factor. *Synapse* 66 630–639. 10.1002/syn.21549 22337473 PMC3824965

[B12] Geva M. Kusko R. Soares H. Fowler K. D. Birnberg T. Barash S. (2016). Pridopidine activates neuroprotective pathways impaired in Huntington disease. *Hum. Mol. Genet.* 25 3975–3987. 10.1093/hmg/ddw238 27466197 PMC5291233

[B13] Hartveit E. Veruki M. L. (2007). Studying properties of neurotransmitter receptors by non-stationary noise analysis of spontaneous postsynaptic currents and agonist-evoked responses in outside-out patches. *Nat. Protoc.* 2 434–448. 10.1038/nprot.2007.47 17406605

[B14] Hayashi T. Su T.-P. (2007). Sigma-1 receptor chaperones at the ER- mitochondrion interface regulate Ca2+ signaling and cell survival. *Cell* 131 596–610. 10.1016/j.cell.2007.08.036 17981125

[B15] Ibata K. Sun Q. Turrigiano G. G. (2008). Rapid synaptic scaling induced by changes in postsynaptic firing. *Neuron* 57 819–826. 10.1016/j.neuron.2008.02.031 18367083

[B16] Ionescu A. Gradus T. Altman T. Maimon R. Saraf Avraham N. Geva M. (2019). Targeting the sigma-1 receptor via pridopidine ameliorates central features of ALS pathology in a SOD1G93A model. *Cell Death Dis.* 10:210. 10.1038/s41419-019-1451-2 30824685 PMC6397200

[B17] Johnston T. H. Geva M. Steiner L. Orbach A. Papapetropoulos S. Savola J.-M. (2018). Pridopidine, a clinic-ready compound, reduces 3,4-dihydroxyphenylalanine-induced dyskinesia in Parkinsonian macaques. *Mov. Disord.* 10.1002/mds.27565 [Epub ahead of print]. 30575996

[B18] Kieburtz K. Reilmann R. Olanow C. W. (2018). Huntington’s disease: current and future therapeutic prospects. *Mov. Disord.* 33 1033–1041. 10.1002/mds.27363 29737569

[B19] Kikuchi-Utsumi K. Nakaki T. (2008). Chronic treatment with a selective ligand for the sigma-1 receptor chaperone, SA4503, up-regulates BDNF protein levels in the rat hippocampus. *Neurosci. Lett.* 440 19–22. 10.1016/j.neulet.2008.05.055 18547721

[B20] Kirov S. A. Sorra K. E. Harris K. M. (1999). Slices have more synapses than perfusion-fixed hippocampus from both young and mature rats. *J. Neurosci.* 19 2876–2886. 10.1523/JNEUROSCI.19-08-02876.1999 10191305 PMC6782277

[B21] Kourrich S. Su T.-P. Fujimoto M. Bonci A. (2012). The sigma-1 receptor: roles in neuronal plasticity and disease. *Trends Neurosci.* 35 762–771. 10.1016/j.tins.2012.09.007 23102998 PMC3587126

[B22] Liang X. Wang R. Y. (1998). Biphasic modulatory action of the selective sigma receptor ligand SR 31742A on N-methyl-d-aspartate-induced neuronal responses in the frontal cortex. *Brain Res.* 807 208–213. 10.1016/s0006-8993(98)00797-5 9757040

[B23] MacDonald M. E. Ambrose C. M. Duyao M. P. Myers R. H. Lin C. Srinidhi L. (1993). A novel gene containing a trinucleotide repeat that is expanded and unstable on Huntington’s disease chromosomes. *Cell* 72 971–983. 10.1016/0092-8674(93)90585-E 8458085

[B24] Marder E. Goaillard J.-M. (2006). Variability, compensation and homeostasis in neuron and network function. *Nat. Rev. Neurosci.* 7 563–574. 10.1038/nrn1949 16791145

[B25] Mendez P. Stefanelli T. Flores C. E. Muller D. Lüscher C. (2018). Homeostatic plasticity in the hippocampus facilitates memory extinction. *Cell Rep.* 22 1451–1461. 10.1016/j.celrep.2018.01.025 29425501

[B26] Milnerwood A. J. Gladding C. M. Pouladi M. A. Kaufman A. M. Hines R. M. Boyd J. D. (2010). Early increase in extrasynaptic NMDA receptor signaling and expression contributes to phenotype onset in Huntington’s disease mice. *Neuron* 65 178–190. 10.1016/j.neuron.2010.01.008 20152125

[B27] Milnerwood A. J. Kaufman A. M. Sepers M. D. Gladding C. M. Zhang L. Wang L. (2012). Mitigation of augmented extrasynaptic NMDAR signaling and apoptosis in cortico-striatal co-cultures from Huntington’s disease mice. *Neurobiol. Dis.* 48 40–51. 10.1016/j.nbd.2012.05.013 22668780

[B28] Milnerwood A. J. Raymond L. A. (2010). Early synaptic pathophysiology in neurodegeneration: insights from Huntington’s disease. *Trends Neurosci.* 33 513–523. 10.1016/j.tins.2010.08.002 20850189

[B29] Orth M. Schippling S. Schneider S. A. Bhatia K. P. Talelli P. Tabrizi S. J. (2010). Abnormal motor cortex plasticity in premanifest and very early manifest Huntington disease. *J. Neurol. Neurosurg. Psychiatry* 81 267–270. 10.1136/jnnp.2009.171926 19828482 PMC2997479

[B30] Plotkin J. L. Surmeier D. J. (2015). Corticostriatal synaptic adaptations in Huntington’s disease. *Curr. Opin. Neurobiol.* 33 53–62. 10.1016/j.conb.2015.01.020 25700146 PMC4831704

[B31] Poo M. (2001). Neurotrophins as synaptic modulators. *Nat. Rev. Neurosci.* 2 24–32. 10.1038/35049004 11253356

[B32] Reilmann R. McGarry A. Grachev I. D. Savola J.-M. Borowsky B. Eyal E. (2018). Safety and efficacy of pridopidine in patients with Huntington’s disease (PRIDE-HD): a phase 2, randomised, placebo-controlled, multicentre, dose-ranging study. *Lancet Neurol.* 18 165–176. 10.1016/S1474-4422(18)30391-0 30563778

[B33] Rutherford L. C. Nelson S. B. Turrigiano G. G. (1998). BDNF has opposite effects on the quantal amplitude of pyramidal neuron and interneuron excitatory synapses. *Neuron* 21 521–530. 10.1016/s0896-6273(00)80563-2 9768839

[B34] Ryskamp D. Wu J. Geva M. Kusko R. Grossman I. Hayden M. (2017). The sigma-1 receptor mediates the beneficial effects of pridopidine in a mouse model of Huntington disease. *Neurobiol. Dis.* 97 46–59. 10.1016/j.nbd.2016.10.006 27818324 PMC5214572

[B35] Ryskamp D. Wu L. Wu J. Kim D. Rammes G. Geva M. (2019). Pridopidine stabilizes mushroom spines in mouse models of Alzheimer’s disease by acting on the sigma-1 receptor. *Neurobiol. Dis.* 124 489–504. 10.1016/j.nbd.2018.12.022 30594810 PMC6363865

[B36] Sahlholm K. Århem P. Fuxe K. Marcellino D. (2013). The dopamine stabilizers ACR16 and (-)-OSU6162 display nanomolar affinities at the σ-1 receptor. *Mol. Psychiatry* 18 12–14. 10.1038/mp.2012.3 22349783

[B37] Sahlholm K. Sijbesma J. W. A. Maas B. Kwizera C. Marcellino D. Ramakrishnan N. K. (2015). Pridopidine selectively occupies sigma-1 rather than dopamine D2 receptors at behaviorally active doses. *Psychopharmacology* 232 3443–3453. 10.1007/s00213-015-3997-8 26159455 PMC4537502

[B38] Saudou F. Humbert S. (2016). The Biology of Huntingtin. *Neuron* 89 910–926. 10.1016/j.neuron.2016.02.003 26938440

[B39] Schippling S. Schneider S. A. Bhatia K. P. Münchau A. Rothwell J. C. Tabrizi S. J. (2009). Abnormal motor cortex excitability in preclinical and very early Huntington’s disease. *Biol. Psychiatry* 65 959–965. 10.1016/j.biopsych.2008.12.026 19200948 PMC2998173

[B40] Shirasaki D. I. Greiner E. R. Al-Ramahi I. Gray M. Boontheung P. Geschwind D. H. (2012). Network organization of the huntingtin proteomic interactome in mammalian brain. *Neuron* 75 41–57. 10.1016/j.neuron.2012.05.024 22794259 PMC3432264

[B41] Slow E. J. van Raamsdonk J. Rogers D. Coleman S. H. Graham R. K. Deng Y. (2003). Selective striatal neuronal loss in a YAC128 mouse model of Huntington disease. *Hum. Mol. Genet.* 12 1555–1567. 10.1093/hmg/ddg169 12812983

[B42] Small D. H. (2008). Network dysfunction in Alzheimer’s disease: does synaptic scaling drive disease progression? *Trends Mol. Med.* 14 103–105. 10.1016/j.molmed.2007.12.006 18262842

[B43] Tang T.-S. Tu H. Chan E. Y. W. Maximov A. Wang Z. Wellington C. L. (2003). Huntingtin and huntingtin-associated protein 1 influence neuronal calcium signaling mediated by inositol-(1,4,x5) triphosphate receptor type 1. *Neuron* 39 227–239. 10.1016/S0896-6273(03)00366-0 12873381 PMC3220623

[B44] Thu D. C. V. Oorschot D. E. Tippett L. J. Nana A. L. Hogg V. M. Synek B. J. (2010). Cell loss in the motor and cingulate cortex correlates with symptomatology in Huntington’s disease. *Brain* 133 1094–1110. 10.1093/brain/awq047 20375136

[B45] Tian X. Kai L. Hockberger P. E. Wokosin D. L. Surmeier D. J. (2010). MEF-2 regulates activity-dependent spine loss in striatopallidal medium spiny neurons. *Mol. Cell. Neurosci.* 44 94–108. 10.1016/j.mcn.2010.01.012 20197093 PMC2878643

[B46] Traynelis S. F. Angus Silver R. Cull-Candy S. G. (1993). Estimated conductance of glutamate receptor channels activated during EPSCs at the cerebellar mossy fiber-granule cell synapse. *Neuron* 11 279–289. 10.1016/0896-6273(93)90184-S 7688973

[B47] Turrigiano G. (2007). Homeostatic signaling: the positive side of negative feedback. *Curr. Opin. Neurobiol.* 17 318–324. 10.1016/j.conb.2007.04.004 17451937

[B48] Turrigiano G. (2011). Homeostatic synaptic plasticity: local and global mechanisms for stabilizing neuronal function. *Cold Spring Harb. Perspect. Biol.* 4:a005736. 10.1101/cshperspect.a005736 22086977 PMC3249629

[B49] Turrigiano G. G. Leslie K. R. Desai N. S. Rutherford L. C. Nelson S. B. (1998). Activity-dependent scaling of quantal amplitude in neocortical neurons. *Nature* 391 892–896. 10.1038/36103 9495341

[B50] Virlogeux A. Moutaux E. Christaller W. Genoux A. Bruyère J. Fino E. (2018). Reconstituting corticostriatal network on-a-chip reveals the contribution of the presynaptic compartment to Huntington’s disease. *Cell Rep.* 22 110–122. 10.1016/j.celrep.2017.12.013 29298414

[B51] Wang J. K. T. Langfelder P. Horvath S. Palazzolo M. J. (2017). Exosomes and homeostatic synaptic plasticity are linked to each other and to Huntington’s, Parkinson’s, and other neurodegenerative diseases by database-enabled analyses of comprehensively curated datasets. *Front. Neurosci.* 11:149 10.3389/fnins.2017.00149 PMC5374209 28611571

[B52] Watt A. J. Desai N. S. (2010). Homeostatic plasticity and STDP: keeping a neuron’s cool in a fluctuating world. *Front. Synaptic Neurosci.* 2:5 10.3389/fnsyn.2010.00005 PMC3059670 21423491

[B53] Wierenga C. J. Walsh M. F. Turrigiano G. G. (2006). Temporal regulation of the expression locus of homeostatic plasticity. *J. Neurophysiol.* 96 2127–2133. 10.1152/jn.00107.2006 16760351

[B54] Zuccato C. Cattaneo E. (2007). Role of brain-derived neurotrophic factor in Huntington’s disease. *Prog. Neurobiol.* 81 294–330. 10.1016/j.pneurobio.2007.01.003 17379385

